# Safety and Efficacy in Mitral Regurgitation Management with the MitraClip^®^ G4 System: Insights from a Single-Center Study

**DOI:** 10.3390/jcdd12010004

**Published:** 2024-12-25

**Authors:** Georgios E. Papadopoulos, Ilias Ninios, Sotirios Evangelou, Andreas Ioannidis, Vlasis Ninios

**Affiliations:** 2nd Cardiology Department, Interbalkan Medical Center, 55535 Thessaloniki, Greece

**Keywords:** mitral regurgitation, transcatheter edge-to-edge repair, MitraClip, MR severity, QoL, NYHA class

## Abstract

Background: Mitral regurgitation (MR) is a common valvular disorder linked to high morbidity and mortality. For patients unsuitable for surgery, transcatheter mitral edge-to-edge repair (TEER) with the MitraClip^®^ G4 system offers an alternative. This study aims to evaluate procedural, echocardiographic, functional, and quality of life (QoL) outcomes in patients who underwent TEER with the MitraClip^®^ G4 system, along with possible predictors of New York Heart Association (NYHA) class I at 30 days and at 1 year. Methods: Patients with moderate-to-severe (3+) or severe (4+) degenerative MR (DMR) or functional MR (FMR), classified as NYHA class III or IV, and who underwent TEER with the MitraClip^®^ G4 system at our center between January 2021 and December 2023 were included. Results: A total of 83 patients [71% FMR, 66% male, median (IQR) age 70 (11) years] underwent TEER, with 100% procedural success. MR ≤ 2+ was achieved in 100% and 98% of patients at 30 days and 1 year, respectively. NYHA class I or II was achieved in 100% and 96.8% of patients at 30 days and 1 year, respectively. The Kansas City Cardiomyopathy Questionnaire (KCCQ) score improved from 51 ± 20 at baseline to 69 ± 15 at 30 days (*p* < 0.001) and 70.5 ± 15 at 1 year (*p* < 0.001). Lower baseline N-terminal pro-brain natriuretic peptide (NT-proBNP) predicted achieving NYHA class I at 30 days (HR: 0.63, 95% CI: 0.41–0.95, *p* = 0.030), while lower European System for Cardiac Operative Risk Evaluation II (EuroSCORE II) and NT-proBNP predicted it at 1 year [(HR: 0.50, 95% CI: 0.28–0.89, *p* = 0.019), (HR: 0.67, 95% CI: 0.44–0.99, *p* = 0.049), respectively]. Conclusions: The MitraClip^®^ G4 system provides significant improvements in MR severity, functional class, and QoL. Lower NT-proBNP and EuroSCORE II were strong predictors of achieving optimal functional status (NYHA class I).

## 1. Introduction

Mitral Regurgitation (MR) stands as the second most common valvular disorder, prevalent in approximately 10% of individuals aged 75 years and above, imposing significant morbidity and mortality burdens [1,2,3,4]. The landscape of MR management has witnessed a paradigm shift with the introduction of transcatheter edge-to-edge repair (TEER) utilizing the MitraClip^®^ system (Abbott Vascular, Santa Clara, CA, USA). While surgical mitral valve repair has historically been the cornerstone for degenerative MR (DMR), TEER has emerged as a promising alternative, particularly for patients who were previously ineligible for surgical intervention [5].

The EVEREST II trial demonstrated promising outcomes in low-risk DMR patients [6], yet with a higher recurrence rate after 4-year results [7]. TEER induction in treating severe functional or secondary mitral regurgitation (FMR) was assessed by the discordant results of the MITRA-FR [8] and COAPT trials [9]. While MITRA-FR failed to demonstrate a significant impact on mortality or heart failure hospitalizations at one year, the COAPT trial illuminated a survival advantage associated with TEER, stimulating ongoing discourse and inquiry into the optimal role of TEER in MR management. Furthermore, the EXPAND G4 study [10] evaluated the one-year outcomes of patients treated with the fourth generation MitraClip^®^ device, drawing conclusions about its safety and efficacy over a one-year period, with a significant reduction in the MR severity to ≤1+ in over 90% of patients. The most recent randomized controlled trial, RESHAPE-HF2, demonstrated that the addition of transcatheter mitral valve repair significantly improved outcomes in patients with moderate-to-severe MR, including a reduction in heart failure-related hospitalizations and improvements in quality of life (QoL) compared to medical therapy alone [11].

This dynamic landscape underscores the need for continued research and collaborative efforts to refine patient selection, optimize outcomes, and elucidate the true potential of TEER in addressing the complexities of MR and its sequelae. This study aims to assess the 1 year outcomes in subjects who underwent treatment with the Mitraclip^®^ G4 system, particularly focusing on its safety and performance in a real-world context of a single-center, single-operator experience.

## 2. Materials and Methods

This retrospective observational cohort study encompasses data extracted from the medical records of all patients who underwent TEER at our structural heart disease expert center from January 2021 to December 2023. The study received approval from the Institutional Review Board of our center, adhering to the principles outlined in the Declaration of Helsinki.

### 2.1. Study Population

All patients diagnosed with moderate-to-severe (3+) or severe (4+) degenerative MR (DMR) or functional MR (FMR), classified as New York Heart Association (NYHA) class III or IV despite the use of stable maximal tolerated doses of guideline-directed medical therapy (GDMT), and who underwent TEER using the MitraClip^®^ G4 system in the Hybrid Operating Room at the Interbalkan Medical Center were included. DMR was defined as MR resulting from a compromised mitral valve, while FMR was characterized as MR stemming from a compromised left ventricle or atria while the valve remained anatomically intact. The decision to perform the transcatheter procedure was made by the local heart team as guided by the 2021 European Society of Cardiology (ESC)/European Association for Cardio-Thoracic Surgery (EACTS) guidelines [12]. Patients in cardiogenic shock or with hemodynamic instability requiring inotropic support and with a life-expectancy of <12 months due to significant non-cardiac comorbidities were excluded.

### 2.2. TEER Procedure

The transcatheter edge-to-edge repair (TEER) procedure was performed under general anesthesia in the Hybrid Operating Room at the Interbalkan Medical Center, utilizing the MitraClip^®^ G4 system (Abbott Vascular, Santa Clara, CA, USA) by a single operator.

Venous access was established via the right femoral vein using a large-bore sheath. A transseptal puncture was performed using a specialized needle to gain access to the left atrium, ensuring an optimal puncture site to facilitate subsequent navigation towards the mitral valve. A steerable guide catheter (SGC) was then advanced into the left atrium under real-time fluoroscopic and transesophageal echocardiographic guidance.

Once the left atrium was accessed, the MitraClip Delivery System (CDS) was carefully maneuvered towards the mitral valve through the SGC. Continuous TEE monitoring was employed to assess the orientation and position of the catheter relative to the mitral leaflets. Precise manipulation of the clip delivery system was necessary to align the MitraClip with the target mitral leaflets. The device was positioned at the site of regurgitation, typically at the A2-P2 scallop, which is the most common location for mitral valve prolapse or flail.

After ensuring optimal positioning and alignment, the clip was opened and advanced to grasp both the anterior and posterior mitral leaflets. The clip arms were closed to approximate the leaflets, creating a double orifice and reducing the regurgitant flow. TEE was used extensively throughout this phase to confirm adequate leaflet capture, ensure proper coaptation, and evaluate the immediate reduction in MR. If the reduction in MR was deemed insufficient or if clip positioning was suboptimal, the clip could be repositioned, or additional clips could be deployed as needed.

After the successful deployment of the MitraClip, a comprehensive echocardiographic assessment was performed to evaluate the final result. This included checking for residual MR, assessing leaflet mobility and adequate leaflet grasping, and confirming that no significant mitral stenosis had been induced. Hemodynamic measurements were repeated to assess the improvement in cardiac function post-procedure.

Following the procedure, patients were transferred to the intensive care unit for close monitoring. Post-procedural TEE was typically performed within 24 h to confirm the stability of the MitraClip and to reassess MR severity.

### 2.3. Definition of the Variables

Baseline characteristics, including age, sex, estimated glomerular filtration rate (eGFR), European System for Cardiac Operative Risk Evaluation II (EuroSCORE II), cardiopulmonary comorbidities (e.g., prior heart failure hospitalization, atrial fibrillation [AF], prior myocardial infarction [MI], diabetes, chronic obstructive pulmonary disease [COPD]), the presence of permanent pacemaker (PPM) or implantable cardioverter defibrillator (ICD), cardiac resynchronization therapy (CRT), N-terminal pro-brain natriuretic peptide (NT-proBNP), and NYHA class were recorded. Transthoracic echocardiography (TTE) was conducted to assess left ventricular ejection fraction (LVEF), left ventricular end-diastolic diameter (LVDD), left ventricular end-systolic diameter (LVSD), effective regurgitant orifice (ERO), and MR severity. MR severity classification comprises of trivial (0+), mild (1+), moderate (2+), moderate-to-severe (3+), and severe (4+). All TTEs were conducted by the same examiner. Right heart catheterization was performed before each procedure to measure mean right atrial pressure (mRAP), pulmonary capillary wedge pressure (PCWP), systolic pulmonary artery pressure (PASP), mean pulmonary artery pressure (mPAP), and cardiac index (CI). MR etiology was classified as DMR or FMR by the heart team. Technical success was defined as the absence of procedural mortality; the successful access, delivery, and retrieval of the device delivery system; the successful deployment and correct positioning of the first intended device; and freedom from emergency surgery or reintervention related to the device or access procedure [13]. Procedural success was defined as a device success of either optimal or acceptable and the absence of major device- or procedure-related serious adverse events, including the following: (i) death, (ii) stroke, (iii) life-threatening bleeding, (iv) major vascular complications, (v) major cardiac structural complications, (vi) stage 2 or 3 acute kidney injury (includes new dialysis), (vii) myocardial infarction or coronary ischemia requiring PCI or CABG, (viii) severe hypotension, heart failure, or respiratory failure requiring intravenous pressors or invasive or mechanical heart failure treatments such as ultrafiltration or hemodynamic assist devices, including intra-aortic balloon pumps, left ventricular or biventricular assist devices, or prolonged intubation for ≥48 h, (ix) any valve-related dysfunction, migration, thrombosis, or other complication requiring surgery or repeat intervention [13].

### 2.4. Study Objectives

A TTE was performed at baseline, 30 day, and 1 year follow-up visits to assess MR severity. NYHA class and Kansas City Cardiomyopathy Questionnaire (KCCQ) scores were also recorded at baseline, 30 day, and 1 year follow-up visits. Survival and hospitalization data were obtained either through telephone contact or via the national electronic health record, when available. The main objectives of the study were to report 1 year mortality and hospitalization rates, to analyze the change in MR severity and geometric distortion of the left ventricle, NYHA class, and QoL throughout follow-up visits, and to identify plausible baseline prognostic factors for NYHA class I classification at 30 days and at 1 year.

### 2.5. Statistical Analysis

Continuous variables following normal distribution were presented as mean and standard deviation (SD), while variables that were not distributed normally were presented as median and interquartile range (IQR). The normality of distribution was assessed by comparing mean and median values, comparing graphical representations of the distribution of the variables, and by using the Kolmogorov–Smirnov test. Qualitative variables were summarized using absolute and relative frequencies [n/N (%)]. Statistical comparisons of continuous variables that exhibited normal distribution were performed using the student *t*-test, while the Wilcoxon rank-sum test was employed for variables that did not follow a normal distribution. Categorical variables were compared with the χ^2^ test or the Fisher exact test if cell counts were small (≤5). The Kruskal–Wallis test was used for the comparison of continuous variables between more than two independent samples. A multivariate Cox proportional hazards model was fitted to identify the independent predictors of NYHA class I in 30 day and 1 year follow-up visits. The purposeful selection process was used to identify candidate variables for our model. Variables with a *p*-value < 0.2 in the univariate analysis were included in our model. The ratio of events to explanatory variables was 10 in order to avoid over-fitting of the model. The application of a Cox proportional hazards model yielded hazard ratios and 95% confidence intervals. The Akaike Information Criterion (AIC) was used to select the model with the best fit. All statistical analyses were performed on RStudio version 2023.03.0+386.

## 3. Results

### 3.1. Baseline Characteristics and Procedural Results

A total of 83 patients (71% FMR, 66% male) underwent TEER, with a median age of 70 years (IQR: 11 years) at baseline. Among patients with FMR, ischemic etiology was present in 63% (37/59), while 37% (22/59) had non-ischemic dilative cardiomyopathy. A comprehensive overview of the baseline characteristics of the patients according to the MR etiology is presented in Table 1. Patients with FMR exhibited a higher prevalence of prior myocardial infarction (MI) and presence of permanent pacemaker (PPM), implantable cardioverter-defibrillator (ICD) or cardiac resynchronization therapy (CRT). Additionally, the FMR group had lower left ventricular ejection fraction (LVEF), lower cardiac index (CI), and larger left ventricular end-diastolic diameter (LVDD). In contrast, patients with degenerative mitral regurgitation (DMR) had larger effective regurgitant orifice (ERO). There was no statistically significant difference in the incidence of severe MR between the two groups.

Overall, the median post-TEER transmitral gradient was 3.53 (IQR: 1.2), showing no significant mitral stenosis and similar results between the FMR and DMR groups. The technical and procedural success rates were 100% and 62.7%, respectively. Implantation and procedural time were lower in the DMR group, with no significant difference reported in the number of clips implanted, MR severity at discharge, or hospitalization days.

### 3.2. One-Year Outcomes

After 1 year of follow-up, 10 patients (12%) were hospitalized due to heart failure, of whom 8 subsequently died, resulting in a 1 year mortality rate of 9.6%.

**Figure 1:**
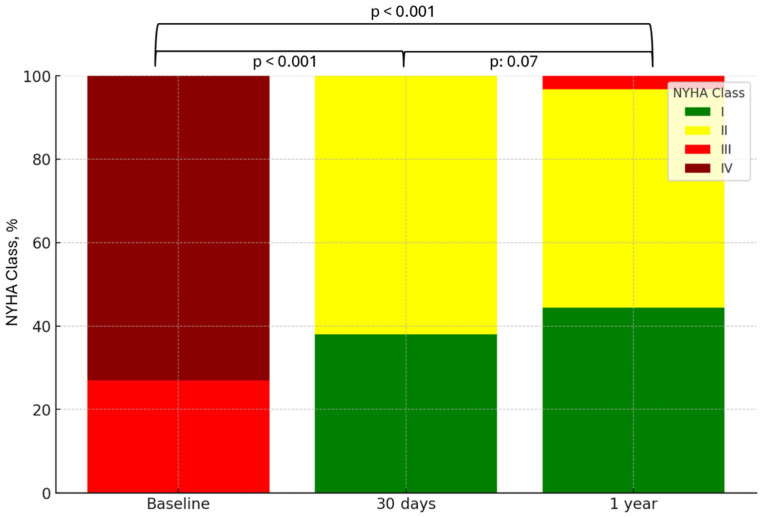
New York Heart Association (NYHA) distribution over time. Abbreviations: NYHA: New York Heart Association.

Figure 1 illustrates the longitudinal distribution of NYHA class among patients. Overall, 31 patients (37%) were asymptomatic (NYHA class I) at 30 days, increasing to 37 patients (44.4%) at 1 year. At the 30 day mark, all patients achieved NYHA class I or II, with 96.8% remaining in this status at 1 year. There was a statistically significant improvement in NYHA class from baseline to both the 30 day and 1 year follow-up visits (*p* < 0.001, *p* < 0.001, respectively).

**Figure 2:**
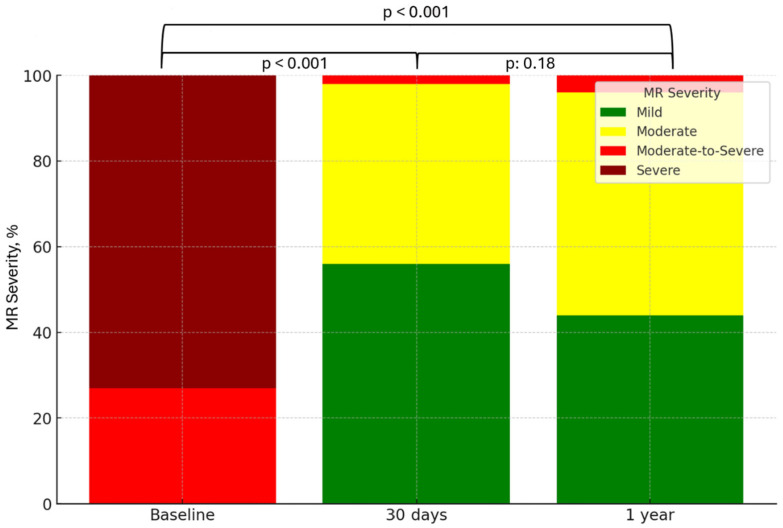
Mitral regurgitation (MR) severity distribution over time. Abbreviations: MR: Mitral Regurgitation.

Figure 2 illustrates the distribution of MR severity over time. At 30 day follow-up visits, no severe MR was reported, and MR ≤2+ was achieved in 81 (98%) patients. At 1 year follow-up visits, MR ≤2+ was achieved in 80 (96%) patients. Notably, 19 (23%) patients that were classified in 4+ MR severity at baseline had only mild MR after 1 year. A substantial improvement in MR severity was reported from baseline to 30 day and 1 year follow-up visits (*p* < 0.001, *p* < 0.001, respectively).

**Figure 3:**
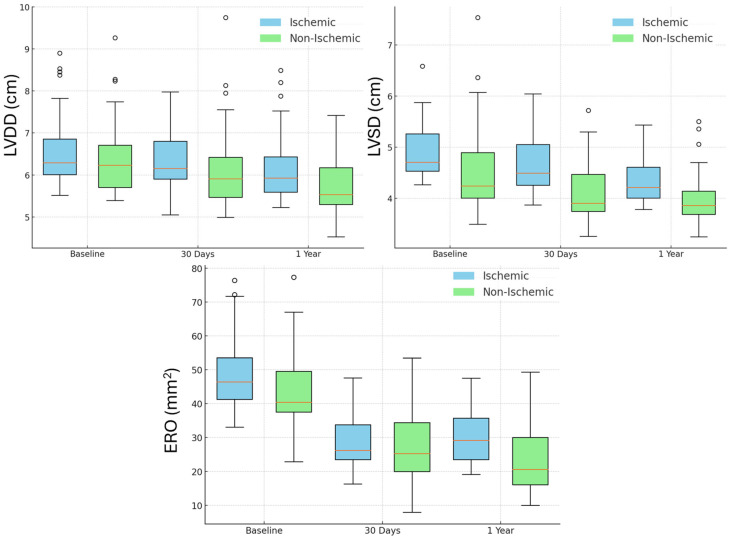
LVDD, LVSD, and ERO distribution over time. Abbreviations: LVDD: Left Ventricular end Diastolic Diameter, LVSD: Left Ventricular end Systolic Diameter, ERO: Effective Regurgitant Orrifice.

Figure 3 illustrates the changes in LVDD, LVSD, and ERO over time. Ischemic FMR patients demonstrated a reduction of LVDD at 30 days [median (IQR): 6.1 (0.5) cm] and at 1 year [5.9 (0.4) cm] compared to baseline [6.4 (0.7) cm; *p*: 0.03 and *p*: 0.04, respectively]. Non-ischemic patients showed a similar reduction at 30 days [5.7 (0.4) cm] and at 1 year [5.5 (0.5) cm] versus baseline [6.0 (0.6) cm; *p*: 0.01 and *p*: 0.02, respectively]. LVESD decreased significantly at 30 days [ischemic: 4.4 (0.4) cm; non-ischemic: 4.0 (0.5) cm] and at 1 year [ischemic: 4.2 (0.4) cm; non-ischemic: 3.8 (0.4) cm] compared to baseline [ischemic: 4.7 (0.6) cm; non-ischemic: 4.3 (0.6) cm; all *p*: 0.04]. ERO was also reduced at 30 days [ischemic: 26 (6) mm^2^; non-ischemic: 21 (7) mm^2^] and at 1 year [ischemic: 25 (5) mm^2^; non-ischemic: 20 (7) mm^2^] versus baseline [ischemic: 44 (10) mm^2^; non-ischemic: 40 (8) mm^2^; all *p* < 0.001].

**Figure 4:**
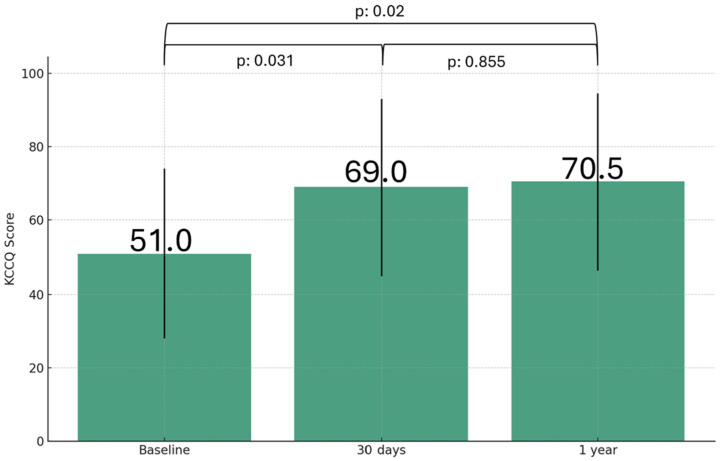
KCCQ score distribution over time. Abbreviations: KCCQ: Kansas City Cardiomyopathy Questionnaire.

Figure 4 illustrates the distribution of KCQQ scores over time. At 30 day and 1 year follow-up visits, there was a statistically significant increase in KCCQ scores compared to baseline values [median (IQR): 69 (53) versus 51 (38); *p* = 0.031 and median (IQR): 70.5 (55) versus 51 (38); *p* = 0.02, respectively].

### 3.3. NYHA Class I Prognostic Factors

Thirty-one (37%) and 37 (44.4%) patients were classified as NYHA class I at 30 days and at 1 year after TEER, respectively. Table 2 presents the cox regression analysis of potential prognostic factors for achieving NYHA class I at 30 days post-TEER. For every unit decrease in the natural logarithm of NT-proBNP values, the likelihood of a patient being classified as NYHA class I at 30 days post-TEER increased by 1.59 times (hazard ratio [HR]: 0.63, 95% confidence interval [CI]: 0.41–0.95, *p* = 0.030).

Table 3 presents the cox regression analysis of potential prognostic factors for achieving NYHA class I at 1 year post-TEER. For every unit decrease in the natural logarithm of EuroSCORE II and NT-proBNP values, the likelihood of a patient being classified as NYHA class I at 1 year post-TEER increased by 2 times (HR: 0.50, 95% CI: 0.28–0.89, *p* = 0.019) and 1.49 times (HR: 0.67, 95% CI: 0.44–0.99, *p* = 0.049), respectively.

## 4. Discussion

Recent findings from the RESHAPE-HF2 trial [11] have further emphasized the role of TEER in improving outcomes for patients with moderate-to-severe FMR and heart failure. This landmark study demonstrated significant reductions in heart failure hospitalizations and improvements in quality of life, reinforcing the importance of addressing MR as a therapeutic target in heart failure management. Consistent with these findings, our single-center study summarizes the 1 year outcomes of patients that underwent TEER for either DMR or FMR and demonstrates a substantial improvement in both quality of life and functional and echocardiographic status, with favorable survival outcomes. Additionally, the study identifies NT-proBNP and EuroSCORE II as potential prognostic indicators for achieving NYHA class I within 30 days or 1 year post-TEER.

In detail, the 1 year mortality rate was 9.6%, which is comparable to those reported from Japan (14.9%) [14], the COAPT trial device group (18.8%) [9], the EXPAND study (14.9%) [15], and the OCEAN-mitral registry (12.3%) [16]. However, higher 1 year mortality rates were observed in the TRAMI (20.2%) [17] (20.2%) and TVT (25.8%) [18] registries, which included larger patient populations. In our study, one-third of the patients were already asymptomatic (NYHA class I) within 30 days after TEER, and NYHA class I/II was achieved by the total cohort. At 1 year, 96.8% achieved NYHA class I/II, which is comparable to the OCEAN-mitral registry (94.1%) [16] and results from Japan (93%) [14], thus higher than the EXPAND study (80.3%) [10]. Similar were the results for the residual MR severity at 1 year, with our study reporting 96% of patients with MR ≤2+, which is higher than the 88.1% reported in the Japanese study [14] and comparable to the 94.1% and 97.5% reported in the OCEAN-mitral registry [16] and the EXPAND study [10], respectively.

This improvement could be predicted by baseline Euroscore II and NT-proBNP values, which were strongly associated with achieving NYHA class I status in 30 days and 1 year. These associations were consistent regardless of MR etiology and residual severity, contrasting with previously established knowledge.

The benefits of the TEER procedure vary between DMR and FMR. In DMR, characterized by intrinsic valvular pathology such as leaflet prolapse or flail, the TEER procedure achieves durable leaflet approximation and MR reduction with minimal impact on left ventricular geometry. Patients often experience significant symptomatic and functional improvements, aligning with findings from pivotal trials like EVEREST II [6]. In contrast, FMR poses greater challenges due to the left ventricular geometry distortions. The COAPT trial [9] demonstrated a survival and quality-of-life advantage for TEER in this population, provided that significant MR reduction is achieved. Importantly, in ischemic FMR, the procedure’s success hinges on appropriate patient selection, with the predictors of benefit including lesser degrees of left ventricular dilation and tethering. This underscores the need for integrating advanced imaging techniques, such as three-dimensional echocardiography, to better delineate leaflet tethering and guide intervention.

Ischemic FMR, a subtype of FMR, often results from left ventricular remodeling after myocardial infarction, particularly involving the displacement of the posterior papillary muscle. This geometric alteration distorts the mitral apparatus, leading to tethering of the mitral leaflets and impaired coaptation. Left ventricular remodeling, including increased sphericity and dilation, exacerbates mitral tethering and increases the regurgitant orifice area, contributing to worse clinical outcomes, such as persistent heart failure symptoms and elevated mortality risk. In ischemic FMR, the posterior papillary muscle displacement is often inferolateral, while in non-ischemic (dilative) FMR, displacement vectors tend to be symmetrical and primarily apical. These distinct displacement patterns reflect differing pathophysiological mechanisms and necessitate tailored therapeutic strategies. The TEER procedure offers a unique advantage in this context by creating a double orifice through leaflet approximation, partially offsetting the adverse effects of tethering and stabilizing the regurgitant zone to restore functional coaptation. However, the extent of left ventricular remodeling post-TEER remains an area of active investigation. Studies have indicated that reverse remodeling, marked by reductions in end-diastolic volume and improved ventricular geometry, may occur following TEER, though the degree of improvement depends on baseline geometric distortion and residual MR [9,10].

In our cohort, non-ischemic FMR patients showed greater relative reductions in LVDD, which may suggest a more favorable reverse remodeling process compared to ischemic FMR patients. This difference could be related to the more symmetrical apical displacement of the papillary muscles in non-ischemic FMR, potentially making them more amenable to volume unloading and leaflet approximation. In contrast, ischemic FMR often involves the asymmetric inferolateral displacement of the posterior papillary muscle, reflecting fixed geometric alterations due to prior myocardial infarction. Despite these differences, both ischemic and non-ischemic subgroups showed significant reductions in EROA, underscoring the efficacy of TEER in improving mitral valve function in both groups. These results highlight the importance of considering the distinct pathophysiological mechanisms in ischemic and non-ischemic FMR when evaluating the effects of TEER and optimizing treatment strategies. Further studies with larger cohorts and longer follow-up periods are needed to validate these findings and explore their long-term clinical implications.

Our study did not assess changes in the anteroposterior diameter of the mitral annulus, tenting volume, or interpapillary distance. These parameters are important components of mitral valve geometry in FMR, and their evaluation could complement our findings. While TEER primarily impacts coaptation length, indirect improvements in annular dimensions and tenting volume have been reported in prior studies as secondary effects of reduced MR and ventricular volume overload. The lack of change in interpapillary distance, however, is expected, given its dependence on left ventricular remodeling. Future studies integrating advanced imaging techniques, such as 3D echocardiography, may better characterize these changes and their clinical significance.

Our study’s primary limitations include a relatively small sample size and a single-center design, potentially restricting the generalizability of our findings. However, it is noteworthy that our center acts as a referral hub for structural heart disease patients across a broader region in northern Greece, mitigating potential selection biases. Moreover, the retrospective nature of our study may introduce biases related to data collection and patient selection. Lastly, our echocardiographic analysis did not include post-procedure measurements of anteroposterior diameter, tenting volume, or interpapillary distance, which limits the ability to fully characterize the geometric effects of TEER. Incorporating these parameters in future studies could provide more comprehensive insights into the structural changes induced by TEER.

## 5. Conclusions

In conclusion, the MitraClip^®^ G4 system proved to be a safe and effective option for treating MR, significantly reducing MR severity and improving both functional status and quality of life, with key baseline markers such as NT-proBNP and EuroSCORE II predicting better clinical outcomes. As the field of transcatheter mitral valve repair continues to evolve, future research should focus on optimizing patient selection and exploring long-term outcomes across broader populations to further enhance the therapeutic potential of this intervention.

## Figures and Tables

**Table 1:** Patients’ baseline characteristics.

Characteristic	Overall, N = 83 ^1^	DMR, N = 24	FMR, N = 59	*p*-Value ^2^
Age, years	76 (11)	79 (9)	75 (11)	0.2
Male sex	55/83 (66)	16/24 (67)	39/59 (66)	>0.9
NYHA class				>0.9
III	22/83 (27)	6/24 (25)	16/59 (27)	
IV	61/83 (73)	18/24 (75)	41/59 (73)	
Euroscore II, %	0.07 (0.06)	0.06 (0.03)	0.08 (0.07)	0.074
eGFR, mL/min/1.73 m^2^	48 (30)	59 (25)	45 (30)	0.12
NT-proBNP, pg/mL	2755 (3351)	1788 (3786)	2876 (3375)	0.072
HF hospitalization	57/83 (69)	17/24 (71)	40/59 (68)	0.8
MI	23/83 (28)	1/24 (4.2)	22/59 (37)	0.002
AF	54/83 (65)	13/24 (54)	41/59 (69)	0.2
PPM	12/83 (14)	0/24 (0)	12/59 (20)	0.015
COPD	31/83 (37)	10/24 (42)	21/59 (36)	0.6
Diabetes	15/83 (18)	5/24 (21)	10/59 (17)	0.8
ICD/CRT				0.023
None	61/83 (73)	22/24 (92)	39/59 (66)	
ICD	11/83 (13)	2/24 (8.3)	9/59 (15)	
CRT	11/83 (13)	0/24 (0)	11/59 (19)	
Echocardiography				
Severe (4+) MR	73/83 (88)	21/24 (88)	52/59 (88)	>0.9
ERO, mm^2^	42 (11)	52 (17)	41 (9)	<0.001
LVEF, %				<0.001
<40	36/83 (43)	3/24 (13)	33/59 (56)	
41–49	30/83 (36)	8/24 (33)	22/59 (37)	
≥50	17/83 (20)	13/24 (54)	4/59 (6.8)	
LVDD, cm	6.10 (1.20)	5.30 (0.75)	6.20 (0.85)	<0.001
LVSD, cm	4.6 (0.8)			
Hemodynamics				
CI, mL/min/m^2^	1.90 (0.60)	2.05 (0.53)	1.80 (0.60)	0.027
PASP, mmHg	55 (15)	53 (20)	55 (15)	0.6
mPAP, mmHg	33 (11)	33 (11)	33 (14)	0.4
PCWP, mmHg	24 (11)	24 (11)	24 (10)	0.4
mRAP, mmHg	10.0 (5.0)	9.5 (6.3)	10.0 (6.8)	0.4
Procedural results				
Technical success	83/83 (100)	24/24 (100)	59/59 (100)	>0.9
Procedural success	52/83 (62.7)	14/24 (58.3)	38/59 (64.4)	0.8
Implantation time, mins	26 (8)	30 (10)	25 (5)	<0.001
Procedural time, mins	55 (11)	63 (10)	52 (7)	<0.001
Number of clips implanted				0.060
1	57/83 (69)	15/24 (63)	42/59 (71)	
2	23/83 (28)	6/24 (25)	17/59 (29)	
3	2/83 (2.4)	2/24 (8.3)	0/59 (0)	
4	1/83 (1.2)	1/24 (4.2)	0/59 (0)	
MR severity at discharge				>0.9
1+ (mild)	6/83 (7.2)	1/24 (4.2)	5/59 (8.5)	
2+ (moderate)	46/83 (55)	13/24 (54)	33/59 (56)	
3+ (moderate-to-severe)	28/83 (34)	9/24 (38)	19/59 (32)	
4+ (severe)	3/83 (3.6)	1/24 (4.2)	2/59 (3.4)	
Transmitral gradient, mmHg	3.53 (1.2)	3.82 (1.3)	3.47 (1.2)	0.3
Hospital length of stay, days	3 (1)	3 (1)	3 (1)	>0.9

^1^ Median (IQR); n/N (%). ^2^ Kruskal-Wallis rank sum test; Pearson’s Chi-squared test; Fisher’s exact test. Note: Continuous variables are presented as median value with interquartile range (IQR). Categorical variables are presented as n/N (%). Abbreviations: DMR: Degenerative Mitral Regurgitation, FMR: Functional Mitral Regurgitation, NYHA: New York Heart Association, eGFR: estimated Glomerular Filtration Rate, NT-proBNP: N terminal-pro brain natriuretic peptide, HF: Heart Failure, MI: Myocardial Infarction, AF: Atrial Fibrillation, PPM: Permanent Pacemaker, COPD: Chronic Obstructive Pulmonary Disease, ICD: Implantable Cardioverter Defibrillator, CRT: Cardiac Resynchronization Therapy, MR: Mitral Regurgitation, ERO: Effective Regurgitant Orifice, LVEF: Left Ventricular Ejection Fraction, LVDD: Left Ventricular End-Diastolic Diameter, CI: Cardiac Index, PASP: Pulmonary Artery Systolic Pressure, mPAP: mean Pulmonary Artery Pressure, PCWP: Pulmonary Capillary Wedge Pressure, mRAP: mean Right Atrial Pressure.

**Table 2:** Adjusted and Unadjusted Hazard Ratios of NYHA class I in 30 days.

	Univariate				Multivariate		
Characteristic	N	HR ^1^	95% CI ^1^	*p*-Value	HR ^1^	95% CI ^1^	*p*-Value
Age	83	1.0	0.96, 1.03	0.7			
Male sex	83	0.91	0.44, 1.90	0.8			
Euroscore II ^2^	83	0.50	0.28, 0.88	0.017	0.64	0.35, 1.14	0.13
eGFR	83	1.01	0.99, 1.03	0.4			
NT-proBNP ^2^	83	0.55	0.37, 0.82	0.003	0.63	0.41, 0.95	0.030
HF Hospitalization	83	0.56	0.27, 1.14	0.11			
MI	83	0.57	0.23, 1.38	0.2			
AF	83	0.82	0.40, 1.68	0.6			
PPM	83	0.35	0.08, 1.47	0.2			
COPD	83	1.77	0.87, 3.58	0.11			
Diabetes	83	0.84	0.32, 2.20	0.7			
ICD/CRT	83	0.90	0.54, 1.50	0.7			
MR etiology	83						
DMR		—	—		—	—	
FMR		0.48	0.23, 0.98	0.044	0.66	0.31, 1.41	0.3
Number of clips implanted	83						
1		—	—				
2		1.34	0.63, 2.86	0.5			
3		5.02	0.67, 37.7	0.12			
MR severity at 30 days	83						
1+		—	—				
2+		1.22	0.59, 2.51	0.6			
3+		1.60	0.21, 12.0	0.6			
MR severity at 1 year	83						
1+		—	—				
2+		1.14	0.40, 3.29	0.8			
3+		0.00	0.00, Inf	>0.9			
Hospital stay	83	0.72	0.37, 1.40	0.3			
Echocardiography							
LVEF	83	5.37	0.16, 182	0.4			
LVDD	83	0.72	0.42, 1.24	0.2			
ERO	83	1.00	0.97, 1.03	>0.9			
Hemodynamics							
PASP	83	0.99	0.96, 1.03	0.6			
PCWP	83	0.98	0.93, 1.03	0.4			
mRAP	83	0.98	0.91, 1.05	0.5			
mPAP	83	0.97	0.93, 1.02	0.3			
CI	83	2.21	1.07, 4.59	0.033			

^1^ HR = Hazard Ratio, CI = Confidence Interval. ^2^ Presented as Natural Logarithm (ln). Abbreviations: DMR: Degenerative Mitral Regurgitation, FMR: Functional Mitral Regurgitation, eGFR: estimated Glomerular Filtration Rate, NT-proBNP: N terminal-pro brain natriuretic peptide, HF: Heart Failure, MI: Myocardial Infarction, AF: Atrial Fibrillation, PPM: Permanent Pacemaker, COPD: Chronic Obstructive Pulmonary Disease, ICD: Implantable Cardioverter Defibrillator, CRT: Cardiac Resynchronization Therapy, MR: Mitral Regurgitation, ERO: Effective Regurgitant Orifice, LVEF: Left Ventricular Ejection Fraction, LVDD: Left Ventricular End-Diastolic Diameter, CI: Cardiac Index, PASP: Pulmonary Artery Systolic Pressure, mPAP: mean Pulmonary Artery Pressure, PCWP: Pulmonary Capillary Wedge Pressure, mRAP: mean Right Atrial Pressure.

**Table 3:** Adjusted and Unadjusted Hazard Ratios of NYHA class I in 1 year.

	Univariate				Multivariate		
Characteristic	N	HR ^1^	95% CI ^1^	*p*-Value	HR ^1^	95% CI ^1^	*p*-Value
Age	83	0.98	0.95, 1.01	0.12			
Male sex	83	0.92	0.45, 1.85	0.8			
Euroscore II ^2^	83	0.40	0.23, 0.69	0.001	0.50	0.28, 0.89	0.019
eGFR	83	1.02	1.00, 1.04	0.025			
NT-proBNP ^2^	83	0.58	0.40, 0.85	0.004	0.67	0.44, 0.99	0.049
HF hospitalization	83	0.49	0.25, 0.96	0.037	0.64	0.32, 1.26	0.2
MI	83	0.49	0.20, 1.18	0.11			
AF	83	0.61	0.31, 1.19	0.15			
PPM	83	0.51	0.16, 1.66	0.3			
COPD	83	1.44	0.73, 2.83	0.3			
Diabetes	83	0.96	0.40, 2.33	>0.9			
ICD/CRT	83	0.89	0.55, 1.46	0.6			
MR etiology	83						
DMR		—	—				
FMR		0.49	0.25, 0.97	0.040			
Number of clips implanted	83						
1		—	—				
2		0.98	0.46, 2.13	>0.9			
3		4.24	0.57, 31.6	0.2			
MR severity at 30 days	83						
1+		—	—				
2+		1.47	0.73, 2.94	0.3			
3+		1.60	0.21, 12.0	0.6			
MR severity at 1 year	83						
1+		—	—				
2+		0.93	0.36, 2.42	0.9			
3+		0.00	0.00, Inf	>0.9			
Hospital stay	83	0.77	0.40, 1.46	0.4			
Echocardiography							
LVEF	83	4.44	0.15, 131	0.4			
LVDD	83	0.79	0.47, 1.32	0.4			
ERO	83	1.00	0.97, 1.03	0.9			
Hemodynamics							
PASP	83	0.99	0.96, 1.02	0.5			
PCWP	83	0.98	0.93, 1.02	0.3			
mRAP	83	0.95	0.88, 1.02	0.14			
mPAP	83	0.97	0.93, 1.02	0.2			
CI	83	1.73	0.87, 3.45	0.12			

^1^ HR = Hazard Ratio, CI = Confidence Interval. ^2^ Presented as Natural Logarithm (ln). Abbreviations: DMR: Degenerative Mitral Regurgitation, FMR: Functional Mitral Regurgitation, eGFR: estimated Glomerular Filtration Rate, NT-proBNP: N terminal-pro brain natriuretic peptide, HF: Heart Failure, MI: Myocardial Infarction, AF: Atrial Fibrillation, PPM: Permanent Pacemaker, COPD: Chronic Obstructive Pulmonary Disease, ICD: Implantable Cardioverter Defibrillator, CRT: Cardiac Resynchronization Therapy, MR: Mitral Regurgitation, ERO: Effective Regurgitant Orifice, LVEF: Left Ventricular Ejection Fraction, LVDD: Left Ventricular End-Diastolic Diameter, CI: Cardiac Index, PASP: Pulmonary Artery Systolic Pressure, mPAP: mean Pulmonary Artery Pressure, PCWP: Pulmonary Capillary Wedge Pressure, mRAP: mean Right Atrial Pressure.

## Data Availability

The data presented in this study are available on request from the corresponding author. The data are not publicly available due to ethical restrictions.
